# Adjunctive Ashwagandha (*Withania somnifera*) Supplementation and Resistance-Training Adaptations in Healthy Adults: A Systematic Review and Random-Effects Meta-Analysis of Muscular Strength, Chest Circumference, and Body Composition

**DOI:** 10.3390/nu18172815

**Published:** 2026-08-27

**Authors:** Jung-Chul Lee, An-Sik Heo

**Affiliations:** Department of Sports Medicine, Dongshin University, Naju 58245, Republic of Korea; channel365@hanmail.net

**Keywords:** *Withania somnifera*, ashwagandha, resistance training, muscular strength, chest circumference, muscle hypertrophy, body composition, dietary supplements, meta-analysis, sports nutrition

## Abstract

**Background/Objectives:** Ashwagandha (*Withania somnifera*) is increasingly marketed as an adjunct that amplifies the strength and muscle-size gains produced by resistance training. Prior meta-analyses pooled trials across mixed exercise contexts rather than trials confined to structured resistance training. We therefore quantified adjunctive ashwagandha effects on muscular strength, chest circumference and body composition within such trials. **Methods:** We searched twelve bibliographic sources (including PubMed/MEDLINE, Europe PMC, OpenAlex and Cochrane CENTRAL) and two trial registers (ClinicalTrials.gov, WHO ICTRP) without date restriction to 23 August 2026, with reference-list, included-study-list and forward-citation screening, for randomized controlled trials (RCTs) combining a standardized ashwagandha extract (root or root-and-leaf) with structured resistance training in healthy adults (PRISMA 2020). Screening, extraction and risk-of-bias assessment were duplicated independently. Change-from-baseline standardized mean differences (SMDs; Hedges *g*) were pooled with restricted maximum-likelihood random-effects models, with RoB 2, leave-one-out and Hartung–Knapp–Sidik–Jonkman (HKSJ) sensitivity analyses, and GRADE. Embase, Scopus, Web of Science and CINAHL were unavailable to us, but Embase- and CINAHL-derived records were retrieved indirectly through CENTRAL. **Results:** Four RCTs were eligible; three (*n* = 161) provided poolable data, and one preprint was reported narratively because its bench-press values were not interpretable as absolute loads. Ashwagandha was associated with greater gains than placebo in 1-RM bench press (SMD 0.60, 95% CI 0.20–1.01), lower-body 1-RM (0.52, 0.19–0.86) and chest circumference (0.86, 0.41–1.31), but not body fat (−0.32, −0.72–0.08). Heterogeneity was low to moderate (*I*^2^ = 7–34%); all three pooled trials raised at least some concerns on RoB 2. Under HKSJ adjustment both strength estimates lost statistical significance and each depended on one influential trial; for chest circumference (k = 2), the HKSJ interval was too imprecise to be informative. GRADE certainty was Low for both strength outcomes and body fat, and Very Low for chest circumference. **Conclusions:** Current evidence does not establish that adjunctive ashwagandha meaningfully enhances resistance-training adaptations; it indicates possible benefits for maximal strength and, indirectly, upper-body girth, but the evidence base is small, statistically fragile and of low to very low certainty, with no dependable effect on adiposity. Adequately powered, independently funded RCTs using direct measures of hypertrophy are required. This review received no external funding and was not registered.

## 1. Introduction

Skeletal muscle strength is a determinant of functional independence, metabolic health, and survival across the lifespan; low muscle strength now anchors the operational definition of sarcopenia, and muscular strength independently predicts all-cause mortality in large general-population cohorts [1,2]. Progressive resistance training is the best-established non-pharmacological stimulus for building and preserving muscle strength and size, and its adaptations are sensitive to modifiable training and nutritional variables [3,4,5,6]. Against this background, interest has grown in nutritional and botanical co-interventions that might augment the strength and hypertrophy produced by resistance training [7,8]. Among these, ashwagandha (*Withania somnifera*), a traditional Ayurvedic adaptogen, has moved rapidly from folk use into mainstream sports-nutrition products marketed explicitly for muscle and strength gains.

Two lines of biological reasoning motivate testing ashwagandha as a resistance-training adjunct. First, its principal bioactive constituents—the withanolides, a family of steroidal lactones—modulate the hypothalamic–pituitary–adrenal (HPA) axis and act on anti-inflammatory and antioxidant targets [9,10,11], and multiple placebo-controlled trials have shown that ashwagandha lowers circulating cortisol relative to placebo [12,13,14], a plausible route to reducing catabolic load during intensive training. Second, ashwagandha may act indirectly on training tolerance by improving sleep quality and perceived recovery [14,15]. At the cellular level, withanolides have been reported to promote myotube differentiation and to attenuate age-related muscle atrophy in rodent models [16,17]. Several small RCTs have since paired ashwagandha with structured resistance training and reported favourable changes in strength and body composition [18,19,20], but individually they are underpowered and heterogeneous in dosing, population, extract type, and outcome definition.

Prior evidence syntheses do not answer the resistance-training question. The existing meta-analytic literature has pooled ashwagandha across broad and mixed “physical-performance” endpoints [21], has focused specifically on aerobic capacity (VO_2_max) [22], or has aggregated heterogeneous performance and cognitive outcomes in broader reviews [23,24]. Muscular strength itself has been meta-analysed: Bonilla et al. reported a medium pooled effect on strength/power outcomes [21], and Zhu et al. recently synthesized physical-function outcomes including strength [24]. These syntheses, however, pooled trials irrespective of whether a structured resistance-training programme was delivered as the co-intervention. What remains unquantified is the narrower question that underpins the product category: within trials that actually delivered a structured, progressive resistance-training programme, the effect of adjunctive ashwagandha on one-repetition-maximum (1-RM) strength of defined movements, on a surrogate index of hypertrophy, and on body composition, tested for robustness and graded for certainty.

We therefore conducted a systematic review and REML random-effects meta-analysis, reported according to PRISMA 2020 [25], of RCTs combining ashwagandha supplementation with structured resistance training in healthy adults. Four outcomes were pre-specified: 1-RM bench press, lower-body 1-RM, chest circumference (a girth surrogate for upper-body hypertrophy), and body-fat percentage. Risk of bias was assessed with RoB 2 [26] and certainty of evidence with GRADE [27,28]. Our aim was not to confirm a marketing claim but to establish, transparently and with an explicit certainty rating, how much—if anything—the current randomized evidence can actually support.

## 2. Materials and Methods

### 2.1. Reporting Guideline Compliance, Registration, and Protocol

**Figure 1:**
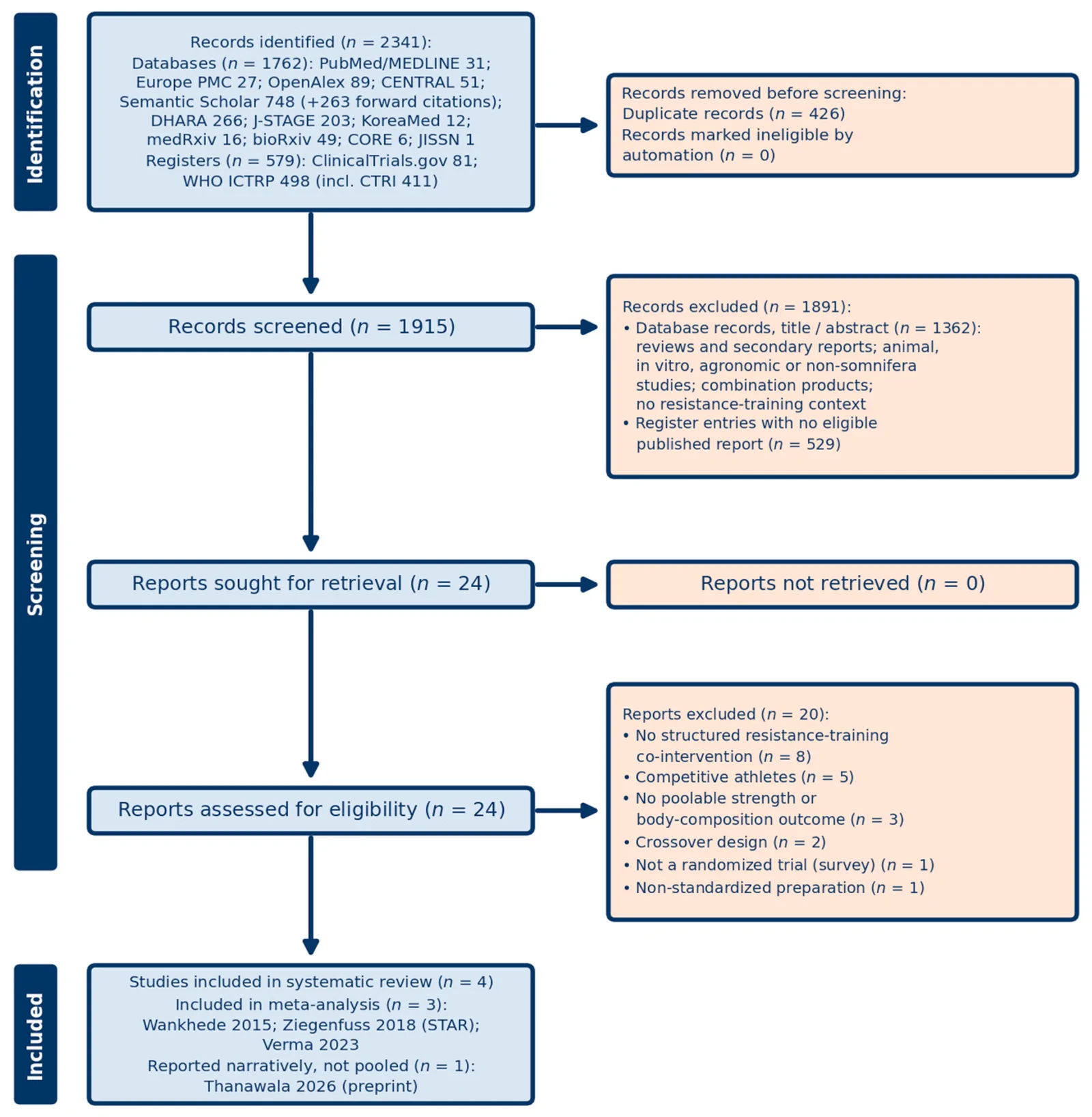
PRISMA 2020 flow diagram of study identification, screening, and inclusion. Of 2341 records identified by the updated and extended searches (final date 23 August 2026; databases *n* = 1762 across twelve sources; registers *n* = 579: ClinicalTrials.gov 81 and WHO ICTRP 498), 426 duplicates were removed and 1915 records were screened; 24 reports were assessed in full text and 20 were excluded for lacking an eligible resistance-training design, population, preparation or poolable outcome. Four randomized controlled trials were eligible; three contributed to the meta-analysis and one preprint is reported narratively because its reported strength values were not interpretable on the required scale. Trials in the inclusion box: Wankhede 2015 [18]; Ziegenfuss (STAR) 2018 [19]; Verma 2023 [20]; reported narratively, Thanawala 2026 [30].

This systematic review and meta-analysis was designed, conducted and reported in full compliance with the Preferred Reporting Items for Systematic Reviews and Meta-Analyses (PRISMA) 2020 statement [25], and followed the methodological conduct standards of the *Cochrane Handbook for Systematic Reviews of Interventions*, version 6.4 [29]. The completed PRISMA 2020 checklist for the main report is provided as Table S1 of the Supplementary Materials, and the completed PRISMA 2020 for Abstracts checklist as Table S2. The PRISMA 2020 flow diagram documenting the identification, screening and inclusion of records is presented as Figure 1 in the main text (Section 3.1).

This review was not prospectively registered in PROSPERO or in any other prospective systematic-review register, and no review protocol was published or otherwise made publicly available before the review was undertaken (PRISMA 2020 items 24a and 24b). The absence of prospective registration is declared here and is carried forward as a limitation of the review process (Section 4.6). To mitigate it, all eligibility criteria, outcomes, effect measures and analytical decisions were fixed before any quantitative synthesis was performed and were documented in an internal, version-controlled analysis plan that is released together with the analysis script (File S1). No amendments were made to that pre-specified plan at any point during the review (PRISMA 2020 item 24c).

The review question was structured with the PICO framework: in healthy adults (Population) undertaking a structured resistance-training programme, does co-administration of a standardized *Withania somnifera* extract (Intervention) versus a matched placebo plus the same training (Comparator) improve muscular strength, muscle size (direct or surrogate measures), and body composition (Outcomes)?

### 2.2. Eligibility Criteria

Studies were eligible if they were parallel-group RCTs enrolling healthy adults; administered an oral, standardized *Withania somnifera* extract—prepared from the root alone or from root and leaf—together with a structured, progressive resistance-training programme; used a matched placebo plus identical training as the comparator; and reported at least one outcome of muscular strength (1-RM), muscle size (direct or anthropometric surrogate measures), or body composition on a scale poolable with the other trials. Reports were excluded if they did not deliver a structured resistance-training co-intervention (for example, high-intensity-interval or endurance protocols, testing-only ergometry, or no exercise at all), used a crossover rather than parallel design, were not randomized, enrolled competitive athletes with non-standardized training, or reported no outcome measured on a common scale. Standardization to withanolide content, rather than the plant fraction used, defined the intervention; accordingly, both the withanolide-standardized root extract (KSM-66) and the standardized root-and-leaf aqueous extract (Sensoril) met this definition. An eligible trial whose reported outcome values were internally implausible—that is, incompatible with the stated measurement scale—was retained in the systematic review but not entered into the quantitative synthesis, and is described narratively (Section 3.1 and Section 3.2). This data-integrity condition was not part of the original eligibility criteria; it was added at the second revision, when the extended search identified a trial to which it applied, and is reported here as a post-hoc criterion. No language or publication-status restriction beyond database indexing was applied; eligibility was initially limited to 2010–2026 to reflect the era of standardized commercial extracts; as an empirical check performed during revision, the PubMed strategy was re-run without any date restriction and retrieved no additional record, and the extended multi-source searches (Section 2.4) identified no eligible trial published before 2010, indicating that the restriction excluded no eligible evidence.

### 2.3. Outcomes

Four outcomes were pre-specified for quantitative synthesis: (i) 1-RM bench press (kg); (ii) lower-body 1-RM (kg), combining leg extension, back squat, and leg press standardized onto a common scale—because all three are direct 1-RM measures obtained with standardized protocols, this pooled estimate was interpreted as an index of broad lower-body maximal strength rather than of any single movement; (iii) chest circumference (cm), treated as an indirect surrogate of upper-body hypertrophy; and (iv) body-fat percentage. For strength and circumference, a positive standardized effect indicated benefit favouring ashwagandha; for body fat, a negative standardized effect (a greater reduction) indicated benefit. Two sources of clinical heterogeneity were noted a priori and carried forward to interpretation: the combination of heterogeneous lower-body movements, and the assessment of body fat by different methods across trials (dual-energy X-ray absorptiometry in one trial, bioelectrical impedance in the others).

### 2.4. Information Sources and Search Strategy

In the original submission, the primary electronic database was PubMed/MEDLINE, searched from 1 January 2010 to 8 July 2026. To reduce the risk of missing eligible trials, the search was supplemented by screening the reference lists of the most relevant prior systematic reviews [21,22,23,24] and by forward-citation checking of the included trials; no additional eligible RCTs were identified from these sources. That original electronic search used title/abstract free-text terms only, without controlled-vocabulary (MeSH) terms and without a study-design filter (design eligibility was applied at screening): (“*Withania somnifera*”[tiab] OR ashwagandha[tiab] OR withanolide*[tiab]) AND (“resistance training”[tiab] OR “strength training”[tiab] OR “resistance exercise”[tiab] OR “weight training”[tiab] OR “muscle strength”[tiab] OR hypertrophy[tiab] OR “body composition”[tiab] OR “1-RM”[tiab] OR “one repetition maximum”[tiab] OR “muscle size”[tiab]).

This strategy returned 29 records (8 July 2026). During revision, the search was updated and extended (final search date 22 August 2026): the PubMed strategy was augmented with controlled-vocabulary terms (“Withania”[Mesh], “Resistance Training”[Mesh], “Muscle Strength”[Mesh]) and re-run without date restriction (31 records); Europe PMC, including preprints (title/abstract fields; 27 records), OpenAlex (title/abstract search; 89 records), and the ClinicalTrials.gov registry (81 records) were searched with equivalent terms; and the reference lists of prior reviews [21,22,23,24], the included-study lists of the meta-analyses of Bonilla et al. [21] and Zhu et al. [24], and forward citations of the included trials were screened. The search was then extended a second time (final search date 23 August 2026) to the Cochrane Central Register of Controlled Trials (CENTRAL; 51 records), the WHO International Clinical Trials Registry Platform (ICTRP; 498 unique trials, of which 411 were registered with the Clinical Trials Registry–India), the Digital Helpline for Ayurveda Research Articles (DHARA; 266 records), Semantic Scholar (748 records, together with 263 forward citations of the included trials), CORE (6 records), J-STAGE (203 records), KoreaMed (12 records), medRxiv and bioRxiv (65 records), and the conference-abstract output of the *Journal of the International Society of Sports Nutrition* (1 record). The included-study lists of the four prior syntheses [21,22,23,24] and of one further 2026 meta-analysis identified during this search were cross-checked item by item against our screening decisions. This second extension identified one additional eligible trial (Section 3.1 and Section 3.2). The full search strings, dates, applied limits and complete record logs for every source are provided as Table S3 of the Supplementary Materials.

### 2.5. Study Selection and Data Extraction

Records were screened in two stages—first on title and abstract, then on full text—against the eligibility criteria. Screening, data extraction, and risk-of-bias assessment were carried out independently and in duplicate by the two authors, with disagreements resolved by discussion until consensus was reached; in addition, every extracted value was re-checked against the source table on a separate pass. The complete extraction dataset (Table S4) and the analysis script (File S1) are provided as Supplementary Materials so that any reader can verify the numbers reported here. For each trial we extracted the sample size per arm, extract type and daily dose, the training programme, and, for each outcome, the mean and standard deviation (SD) of the change from baseline in each arm. Values were read verbatim from the published results tables; data available only as figures, or reported solely as medians with interquartile ranges, were not imputed [31,32]. Where a trial reported the standard error (SE) of the between-group difference in change rather than a per-arm change SD, the common change-score SD was recovered algebraically as SD = SE_diff_ × √(n_1_·n_2_/(n_1_ + n_2_)); for the two equal-sized arms of the STAR trial (*n* = 19 per arm) this reduces to SD = SE_diff_ × √(n/2), giving a change-score SD of 1.76% for body fat from the reported between-group SE of 0.57%. This algebraic recovery is a transformation of a reported statistic, not an imputation of missing data from figures or medians, and Table S4 labels the STAR body-fat change SD as recovered from the reported between-group SE. As a final step before synthesis, the reported values of every eligible trial were checked for internal plausibility against the stated measurement scale; a trial failing this check was reported narratively rather than pooled (Section 3.1).

### 2.6. Effect Measures and Data Transformation

The effect measure was the change-from-baseline standardized mean difference expressed as Hedges *g*, computed by dividing the between-group difference in mean change by the pooled SD and applying the small-sample bias correction J = 1 − 3/(4·*df* − 1) [33,34]. All three included trials reported the SD of the change from baseline directly, or an SE from which it could be recovered (Section 2.5); consequently, no assumption about the pre–post correlation was required, and no correlation-based imputation—or associated sensitivity analysis—was necessary for this synthesis. Had a trial instead reported only baseline and final SDs, the change-score SD would have been derived as √(SD_base_^2^ + SD_final_^2^ − 2·r·SD_base_·SD_final_) under an assumed correlation r, but this situation did not arise.

### 2.7. Statistical Synthesis

For each outcome, effect sizes were pooled with a random-effects model [35,36] using the restricted maximum-likelihood (REML) estimator of the between-study variance τ^2^ [37], implemented in a purpose-written, fully scripted and deterministic analysis pipeline, written in Python 3.12.3 using the NumPy 2.4.4 and SciPy 1.17.1 libraries, that follows the estimators and formulae given in references [33,34,35,36,37,38,39,40,41,42,43]. The complete script is released as File S1 of the Supplementary Materials, so that every estimate reported here can be reproduced directly or re-run independently in any standard meta-analysis package (for example, *metafor* [38]). The full numeric output of all sensitivity and robustness analyses is tabulated in Table S5. For each pooled estimate we report the summary SMD with its 95% confidence interval (CI), a Wald z test, Cochran’s Q with its *p*-value, the *I*^2^ statistic [39,40], and τ^2^.

Because CIs from the conventional model can be anti-conservative when few studies are pooled, a Hartung–Knapp–Sidik–Jonkman (HKSJ) small-sample-adjusted CI was computed as a pre-specified sensitivity analysis for outcomes with at least three studies; at *k* = 2 the HKSJ multiplier is the 97.5th percentile of the t-distribution on one degree of freedom (≈12.71); the resulting interval is mathematically defined but too wide to carry interpretable information, and it is therefore not reported as a meaningful estimate [42,43]. For the same reason, prediction intervals are not reported for any outcome: with *k* ≤ 3 studies τ^2^ is estimated with very low precision, and the t-based multiplier appropriate to a prediction interval (*df* = *k* − 2, that is, *df* = 1 at *k* = 3) yields an interval that carries no interpretable information [41]. Reporting a prediction interval based instead on a normal approximation would understate its true width and was therefore avoided.

Robustness was examined with leave-one-out analyses—reported for the two-study outcome for completeness only, because each removal there leaves a single trial and cannot inform the robustness of a pooled effect—and, for body fat, a pre-specified sensitivity analysis excluding a trial whose reported change-score SD was implausibly small; a fixed-effect model was also fitted to gauge sensitivity to the choice of variance model. Because every outcome pooled fewer than ten trials, funnel-plot asymmetry and regression-based tests for small-study effects, including the Egger test, are uninformative at this number of studies and were therefore not performed [44,45]; publication bias could accordingly not be assessed. Statistical significance was set at two-sided *p* < 0.05.

In accordance with journal policy on generative artificial intelligence, we disclose that a generative artificial intelligence tool (Claude Opus 4.8; Anthropic, San Francisco, CA, USA) assisted in writing the plotting code that renders the pooled estimates as the figures presented below. The estimates themselves were produced by the analysis pipeline described above and were verified by both authors against the source publications; the tool was not used to generate data, to select or extract studies, or to make analytical or interpretive decisions.

### 2.8. Risk of Bias and Certainty of Evidence

Risk of bias in each RCT was assessed with the Cochrane RoB 2 tool across its five domains—randomization process, deviations from intended interventions, missing outcome data, measurement of the outcome, and selection of the reported result [26]—independently by both authors, with disagreements resolved by consensus (Section 2.5); domains with insufficient reporting were rated “some concerns”. The certainty of evidence for each outcome was rated with GRADE [27,28], considering risk of bias, inconsistency [46], indirectness [47], imprecision [48], and publication bias [49], and is summarized in an evidence profile in the Results (Section 3.7).

### 2.9. Study Size and Ethics

As a synthesis of existing trials, no a priori sample-size calculation was performed; the available evidence comprised a small number of small RCTs (total *N* = 123–161 across outcomes), a limitation reflected in the GRADE imprecision assessment. This study analysed aggregate data from previously published RCTs and did not involve new human participants; institutional review board approval and informed consent were therefore not required, and each included trial reported its own ethical approvals in its source publication.

## 3. Results

### 3.1. Study Selection

The original PubMed/MEDLINE search returned 29 records (8 July 2026). Across the first and second extensions (final search date 23 August 2026; Section 2.4), 2341 records were identified: 1762 database records (PubMed/MEDLINE 31; Europe PMC 27; OpenAlex 89; CENTRAL 51; Semantic Scholar 748 together with 263 forward citations; DHARA 266; J-STAGE 203; KoreaMed 12; medRxiv 16; bioRxiv 49; CORE 6; conference-abstract output of the *Journal of the International Society of Sports Nutrition* 1) and 579 register records (ClinicalTrials.gov 81; WHO ICTRP 498 unique trials, including 411 from the Clinical Trials Registry–India). Removal of 426 duplicates left 1915 records for screening. Cross-checking against the included-study lists of the prior syntheses [21,22,23,24] and of one further 2026 meta-analysis identified eight trials we had not previously assessed; all eight were ineligible, having delivered no structured resistance-training co-intervention, enrolled competitive athletes, administered combination products, or used non-standardized preparations. After title/abstract screening, 1891 records were excluded—1362 database records (reviews, editorials, theses and secondary or duplicate reports; animal, in vitro, agronomic or non-somnifera studies; combination products; and human studies without a resistance-training context) and 529 register entries, none of which corresponded to an additional eligible published report—leaving 24 reports for full-text assessment. Twenty were excluded with reasons: eight had no structured resistance-training co-intervention (a high-intensity interval-training trial [50], a team-sport pre-season stress trial [51], an enriched-milk sleep trial [52], an acute-recovery trial, a conference report of self-directed training, and three trials that measured performance without prescribing a training programme); five enrolled competitive athletes with non-standardized training (ref. [53], a wrestling trial, an eight-day handball trial, a national-team wrestling trial and an elite-cyclist trial); three reported no poolable strength or body-composition outcome, including a trial that used cycle-ergometer testing rather than a training programme [54] and a companion report of an included trial that reported only hormonal and inflammatory outcomes; two used a crossover design (ref. [55] and an acute dose–response report); one was a survey rather than a randomized trial; and one administered a non-standardized root powder rather than a standardized extract. Full citations and reasons for all reports excluded at full text are listed in Table S3. Four parallel-group RCTs met all inclusion criteria. Three of them—Wankhede 2015 [18], Ziegenfuss (STAR) 2018 [19], and Verma 2023 [20]—reported outcomes poolable on a common scale and formed the quantitative synthesis. The fourth, a 2026 preprint of a 90-day trial of a sustained-release root extract [30], satisfied every eligibility criterion but reported bench-press one-repetition-maximum values that are not interpretable as absolute loads (Section 3.2); it is therefore described narratively and was not pooled. The PRISMA 2020 flow is shown in Figure 1.

### 3.2. Study Characteristics

The included trials were parallel-group RCTs published between 2015 and 2023 (Table 1). Standardized ashwagandha extracts were administered at 500–600 mg/day for 8–12 weeks. Populations comprised healthy adults: Wankhede 2015 [18] and Ziegenfuss 2018 [19] enrolled men, whereas Verma 2023 [20] enrolled both men and women across multiple centres. Two trials used the withanolide-standardized KSM-66 root extract and one used the root-and-leaf Sensoril aqueous extract, a source of clinical heterogeneity in the intervention. Body fat was measured by dual-energy X-ray absorptiometry in the STAR trial and by bioelectrical impedance in the other two, a measurement difference carried forward to interpretation. All three trials were funded or supplied by ashwagandha manufacturers. The number of participants contributing to each pooled outcome ranged from 123 (*k* = 2) to 161 (*k* = 3). A fourth eligible trial [30] is described here but was not pooled. This 90-day, parallel-group, double-blind, placebo-controlled preprint randomized 84 recreationally active adults aged 25–50 years with a body-mass index of 25.0–29.9 kg/m^2^ and less than one month of strength-training experience (83 completers) to a sustained-release Withania somnifera root extract (300 mg/day, standardized to not less than 4% total USP withanolides) or matched placebo, with both arms following the same supervised, structured and progressive programme of three resistance and two aerobic sessions per week designed in line with National Strength and Conditioning Association recommendations. The primary outcome was the change in bench-press 1-RM, reported as 15.31 kg with the extract versus 7.33 kg with placebo at day 91. The trial was not pooled because its reported 1-RM values are not interpretable as bench-press loads: the published figure places the mean baseline 1-RM at approximately 5.9 kg in both arms, which is below the mass of an unloaded barbell and an order of magnitude smaller than both the baseline loads and the training-induced gains reported in the three pooled trials, and 1-RM was estimated from submaximal repetitions using a prediction equation rather than measured directly. Change-score standard deviations were in any case available only as figure error bars, the report has not been peer reviewed, and it was authored by employees of the manufacturer, so a formal risk-of-bias rating was not assigned. The trial is reported here in full and its implications are considered in Section 4.6.

### 3.3. Risk of Bias

**Figure 2:**
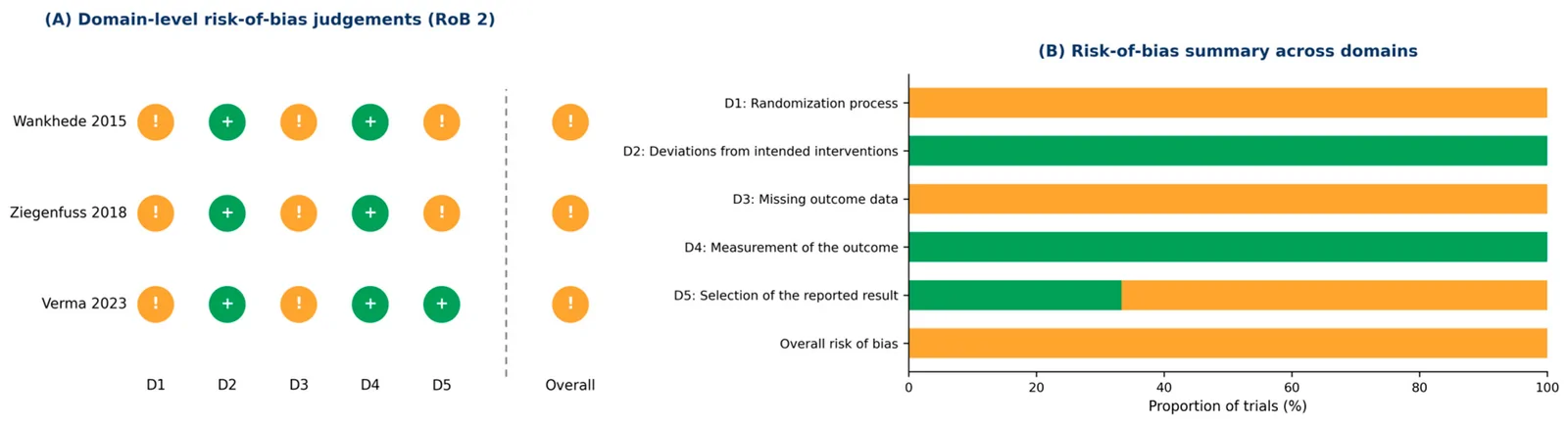
Risk of bias in the included randomized trials assessed with the Cochrane RoB 2 tool: (**A**) domain-level traffic-light judgements and (**B**) the weighted summary across domains. D1–D5 denote the five RoB 2 domains. All three pooled trials were rated as raising some concerns overall. Trials: Wankhede 2015 [18]; Ziegenfuss 2018 [19]; Verma 2023 [20]. In panel (**A**), a green plus sign denotes low risk of bias and a yellow exclamation mark denotes some concerns.

All three pooled trials were judged to raise at least some concerns overall (Figure 2); the fourth eligible trial was not formally rated because it did not enter the quantitative synthesis (Section 3.2). Wankhede 2015 [18] and Ziegenfuss 2018 [19] were each rated as raising “some concerns”, driven by incompletely described allocation concealment (domain 1), completer-only analysis with limited reporting of dropouts (domain 3), and the absence of verifiable prospective outcome registration (domain 5); the outcome-measurement domain (domain 4) was rated low in both, as 1-RM and body composition were assessed with standardized instruments.

Verma 2023 [20] had several methodological strengths—computer-generated stratified randomization in permuted blocks, sealed allocation envelopes, prospective registration with the Clinical Trials Registry of India, and complete outcome reporting, so that domain 5 was rated low—but was nonetheless judged to raise some concerns overall. Domain 1 was rated “some concerns” because baseline values differed significantly between arms for chest circumference (90.0 ± 5.2 vs. 86.4 ± 6.2 cm; *p* = 0.002), mid-arm circumference (*p* = 0.029) and body-mass index (*p* = 0.049); such a pattern raises concern about the randomization process and is directly relevant here, because chest circumference is one of the pooled outcomes. Domain 3 was rated “some concerns” because seven randomized participants (three ashwagandha, four placebo) were excluded from the efficacy analysis for poor compliance, so that the reported analysis was a modified rather than a full intention-to-treat comparison. The trial authors themselves addressed the baseline imbalance by adjusting for sex, body-mass index and baseline chest circumference in an analysis of covariance. The present synthesis, however, required change-from-baseline means and SDs on a metric common to all three trials, so the unadjusted change scores reported in that trial’s results table were used. The resulting potential for residual confounding of the chest-circumference estimate is carried forward to the limitations (Section 4.6). Domain-level and overall judgements are shown in Figure 2.

### 3.4. Primary Syntheses

**Figure 3:**
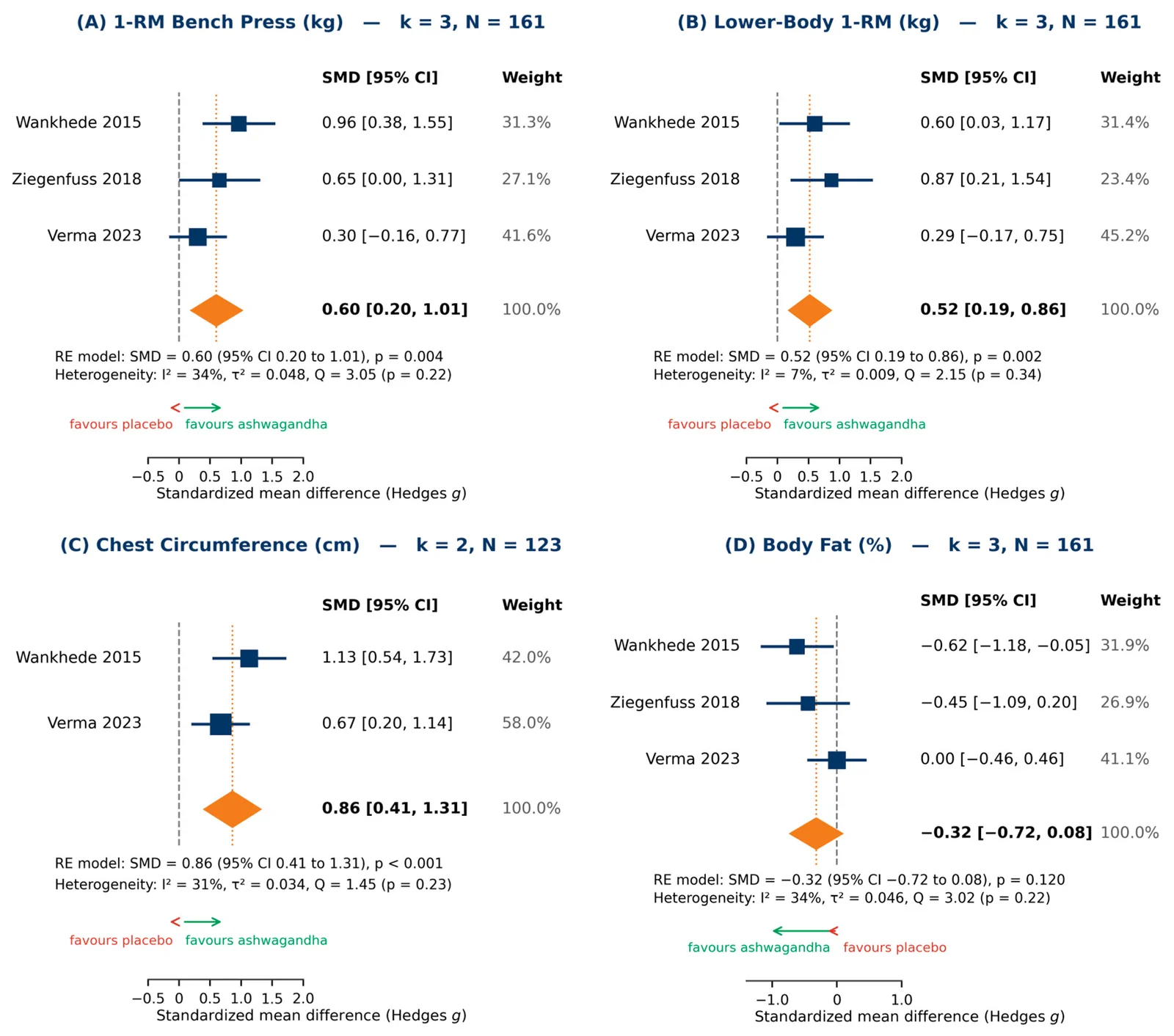
Forest plots of pooled change-from-baseline standardized mean differences (Hedges *g*, random-effects REML model) for (**A**) 1-RM bench press, (**B**) lower-body 1-RM, (**C**) chest circumference, and (**D**) body-fat percentage. Squares are study-level estimates with area proportional to random-effects weight; horizontal lines are 95% confidence intervals; the diamond is the pooled estimate. For body fat, a negative SMD favours ashwagandha. Studies: Wankhede 2015 [18]; Ziegenfuss 2018 [19]; Verma 2023 [20]. In each panel, the grey dashed vertical line marks the null effect (SMD = 0) and the orange dotted line marks the pooled estimate; pooled estimates are set in bold to distinguish them from study-level estimates.

Pooled change-score standardized mean differences (Hedges *g*, REML random-effects) are shown in Figure 3 and Table 2. For 1-RM bench press, the pooled SMD was 0.60 (95% CI 0.20 to 1.01; *p* = 0.004; *I*^2^ = 34.3%; *k* = 3, *n* = 161), favouring ashwagandha. For lower-body 1-RM, the pooled SMD was 0.52 (95% CI 0.19 to 0.86; *p* = 0.002; *I*^2^ = 6.9%; *k* = 3, *n* = 161). For chest circumference, the pooled SMD was 0.86 (95% CI 0.41 to 1.31; *p* < 0.001; *I*^2^ = 30.8%; *k* = 2, *n* = 123)—the largest but least direct estimate, contributed by only two trials. For body-fat percentage, the pooled SMD was −0.32 (95% CI −0.72 to 0.08; *p* = 0.120; *I*^2^ = 33.8%; *k* = 3, *n* = 161), which was not statistically significant; as Section 3.6 shows, however, this null was unstable and depended on a single trial.

### 3.5. Heterogeneity and Robustness

Between-trial heterogeneity was low to moderate for every outcome (*I*^2^ 6.9–34.3%; τ^2^ 0.009–0.048; all Cochran Q *p* > 0.20), and the direction of effect was consistent across trials for the strength outcomes. Robustness was nonetheless limited. Under the small-sample-adjusted HKSJ model, the 95% CI crossed the null for both three-study strength outcomes (bench −0.24 to 1.45; lower-body −0.20 to 1.24), indicating that their conventional significance is not preserved once the small number of studies is properly penalized; for chest circumference, with only two trials, the HKSJ interval is mathematically defined but so wide as to be uninformative (the *k* = 2 multiplier is ≈12.71), so small-sample-adjusted significance could not be assessed for that outcome. Leave-one-out analysis identified an influential trial for each strength estimate: bench-press significance was retained after omitting the largest single estimate (Wankhede 2015 [18]: 0.42 [0.04, 0.80]) but lost after omitting Ziegenfuss 2018 [19] (0.61 [−0.03, 1.25]); lower-body significance was lost after omitting Wankhede 2015 [18] (0.53 [−0.04, 1.10]); and chest circumference remained significant after either single-trial removal (0.67 [0.20, 1.14]; 1.13 [0.54, 1.73]); with only two contributing trials, however, each removal leaves a single study, so these values are reported for completeness and cannot be read as evidence of robustness.

### 3.6. Sensitivity Analyses

In pre-specified sensitivity analyses, the body-fat result proved unstable. Verma 2023 [20] reported a change-from-baseline SD of 0.3% for body fat in both arms; this value is smaller than the documented measurement error of the bioelectrical-impedance method and was judged by both reviewers to be implausibly precise. Questions about measurement precision, and about the reporting of technical measurement error for anthropometric outcomes, were also raised during that article’s published open peer review [20]. Excluding that trial, the pooled body-fat SMD moved from −0.32 (*p* = 0.120) to −0.54 (95% CI −0.97 to −0.12; *p* = 0.013; *I*^2^ = 0%), a statistically significant fat-loss benefit produced simply by removing one high-weight data point whose reported dispersion is not credible. The primary body-fat null should therefore be read as genuinely inconclusive rather than as evidence of no effect. By contrast, refitting all four outcomes with a fixed-effect model changed each pooled estimate by no more than 0.03 SMD (for example, bench press 0.58 vs. 0.60; chest 0.85 vs. 0.86), indicating robustness to the choice of variance model. Small-study effects and publication bias could not be assessed, because every outcome pooled fewer than ten trials (Section 2.7). We did not obtain a corrected change-score SD from the trial authors before completing this revision; the primary analysis therefore retains the published values, and exclusion of that trial remains a sensitivity analysis rather than a data correction.

### 3.7. Certainty of Evidence

Under the GRADE framework, the certainty of evidence was Low for 1-RM bench press, lower-body 1-RM and body fat, and Very Low for chest circumference (Table 3). All four outcomes were downgraded one level for risk of bias, because all three contributing trials raised at least some concerns, and one level for imprecision, reflecting few small trials, a total *N* well below the optimal information size and—for the three-study outcomes—HKSJ intervals crossing the null. No outcome was downgraded for inconsistency, as *I*^2^ was low to moderate and Cochran’s Q was non-significant throughout. Chest circumference was downgraded a third level for indirectness: girth is only a surrogate for muscle hypertrophy, and in the larger of the two contributing trials baseline chest circumference differed significantly between arms (Section 3.3), so its certainty is Very Low. The body-fat synthesis was additionally weakened by measurement heterogeneity (DEXA in one trial, impedance in two) and by the instability documented in Section 3.6; these are discussed but did not lower an already Low rating further. Publication bias could not be formally assessed. The full evidence profile is presented in Table 3.

## 4. Discussion

### 4.1. Principal Findings

This systematic review and REML random-effects meta-analysis synthesized three RCTs (*n* = 161) that combined a standardized ashwagandha extract with a structured, progressive resistance-training programme in healthy adults. Across the three strength- and girth-related outcomes, adjunctive ashwagandha was associated with a statistically significant advantage over placebo: the pooled change-score SMD was 0.60 for 1-RM bench press, 0.52 for lower-body 1-RM, and 0.86 for chest circumference. By conventional benchmarks [56] the two strength estimates are moderate and the girth estimate is the largest of the three, though it was contributed by only two trials. In contrast, the effect on body-fat percentage did not reach significance (−0.32; 95% CI −0.72 to 0.08). Between-trial heterogeneity was low to moderate throughout (*I*^2^ = 7–34%), and every trial pointed in the same direction for the strength outcomes. The central message, however, is not the point estimates but their fragility: neither strength estimate survived the small-sample-adjusted HKSJ model with its significance intact and each depended on an influential trial, while the chest-circumference estimate could not be meaningfully assessed under HKSJ at *k* = 2, and even the body-fat null reversed when a single over-precise data point was removed. The evidence is therefore best summarized as a consistent but Low-certainty signal of benefit for strength, a larger but only indirectly measured and Very-Low-certainty signal for upper-body girth, and no dependable conclusion about adiposity.

### 4.2. Mechanistic Interpretation

Two biologically plausible pathways could underlie the observed associations, though the present data establish neither as causal. First, the withanolides modulate the HPA axis, and multiple placebo-controlled trials have shown that ashwagandha lowers circulating cortisol by roughly a quarter relative to placebo [12,13,14]; a lower catabolic tone during a period of intensive progressive-overload training is a coherent, if indirect, route to greater net anabolism. Second, ashwagandha has anti-inflammatory and antioxidant actions [9,10,11], and the anchor trial reported an attenuated exercise-induced rise in serum creatine kinase alongside its strength effects [18], consistent with reduced muscle damage and faster recovery between sessions. These clinical observations are paralleled by preclinical work in which withanolides promote myotube differentiation and attenuate age-related muscle atrophy [16,17,57], and a withanolide has been shown to inhibit adipogenesis in vitro [58], offering a candidate mechanism for the body-composition signal. A third, equally plausible explanation is that any benefit is mediated by improved sleep and perceived recovery [14,15] rather than by a direct myotrophic action. Crucially, resistance training itself is the proximate and dominant driver of the adaptations measured here [3,4,5,59]; whatever ashwagandha contributes is, at most, an adjunctive amplification of a stimulus that already produces the large majority of the effect. The magnitude of the pooled estimates should be read in that light, and the mechanistic account treated as hypothesis rather than established pathway.

### 4.3. Comparison with Prior Literature

Our findings are directionally consistent with, but narrower and more decision-relevant than, the existing syntheses. Bonilla et al. pooled 13 trials across broad physical-performance endpoints and reported a medium effect on strength/power (d ≈ 0.68) and a very large effect on aerobic capacity [21], while Pérez-Gómez et al. focused on VO_2_max and found a mean increase of about 3 mL·kg^−1^·min^−1^ [22], consistent with individual placebo-controlled trials of ashwagandha and cardiorespiratory endurance [60,61]; more recent syntheses reached similar qualitative conclusions across performance and cognitive outcomes [23,24]. The distinctive contribution of the present review is that it isolates the resistance-training use-case (1-RM strength of defined movements and a hypertrophy surrogate within trials that actually delivered structured resistance training) rather than aggregating mixed performance outcomes. That focus is the source of both our narrower scope and our smaller evidence base: we excluded a fourth ashwagandha RCT that evaluated cycle-ergometer performance, because it used ergometry for testing rather than delivering a structured resistance-training programme [54]. Consistent with subgroup observations in the broader literature, the largest single-trial effects came from the trial in previously untrained men [18], whereas the multicentre trial in already active men and women produced the smallest, tightest estimates [20]. This pattern is consistent with the well-documented dependence of resistance-training responses on training status and age [62], and it implies that the adjunctive benefit, if real, is likely to shrink in trained populations where the training stimulus is already near-maximal.

### 4.4. Clinical and Practical Implications

The practical message is deliberately conservative. Although three of four outcomes reached conventional significance, the certainty of evidence is Low for the two strength outcomes and for body fat and Very Low for chest circumference; the strength signals dissolve under small-sample-adjusted inference and are fragile to single-trial removal; and there was no dependable effect on body composition. To place the strength estimate on a scale that practitioners recognize, an SMD of 0.60 corresponds to approximately 4 kg of additional 1-RM bench-press gain (roughly 1 to 6 kg across the confidence limits) when translated using the placebo-arm change-score SD of the largest included trial (6.3 kg [20]). This translation is illustrative only, because the contributing trials differed several-fold in their change-score dispersion. Under GRADE, Low- and Very-Low-certainty evidence cannot support a strong clinical or public-health recommendation [27,28,48]. A defensible framing for practitioners is that adjunctive ashwagandha *may* confer a modest strength benefit in healthy people who are already resistance training, but that the training itself remains the evidence-based intervention; the supplement is a possible adjunct, not a substitute, and its plausible benefit is smaller than the resistance-training effect it would accompany. Two further considerations temper enthusiasm. First, all of the included trials were funded or supplied by ashwagandha manufacturers, and industry sponsorship is associated with more favourable results [63,64,65]. Second, although ashwagandha is generally well tolerated, a documented case series of ashwagandha-associated liver injury [66] means that even a possible ergogenic benefit must be weighed against a real, if uncommon, safety signal, notwithstanding otherwise reassuring tolerability data [67]. In this context, better-established co-interventions such as adequate protein intake [7,68], creatine [69], and β-hydroxy-β-methylbutyrate [70] rest on far stronger evidence and should take priority in counselling.

### 4.5. Strengths

This review has several methodological strengths. Screening, data extraction and risk-of-bias assessment were performed independently and in duplicate by two reviewers, with disagreements resolved by consensus. Outcomes were pre-specified and deliberately confined to the resistance-training context, so that the pooled estimates answer the question the product category actually raises. Change-from-baseline SMDs were computed with the small-sample bias correction and pooled with an REML random-effects model, and because all included trials reported change-score dispersion directly, the estimates do not depend on an assumed pre–post correlation. The full apparatus of PRISMA 2020, RoB 2 and GRADE was applied through a scripted pipeline that is released with this article, and robustness was interrogated from several complementary angles—leave-one-out removal, a small-sample-adjusted HKSJ model, a body-fat sensitivity analysis excluding an implausibly precise estimate, and a fixed-effect comparison. Analyses that would have been uninformative at this number of studies—regression-based tests for small-study effects and prediction intervals—were withheld rather than reported with caveats. Reporting the fragility of the estimates as prominently as their magnitude is itself a strength and distinguishes this synthesis from more optimistic prior appraisals.

### 4.6. Limitations

Several limitations temper these conclusions. First, the evidence base is very small—two or three trials per outcome and a total of 161 primary participants—well below the optimal information size, so the estimates are imprecise; with k ≤ 3, the between-study variance is estimated so poorly that prediction intervals could not meaningfully be reported at all. Second, the strength estimates are statistically fragile: their conventional significance is not preserved under the HKSJ model, and bench-press and lower-body significance each depend on a single trial. Third, chest circumference—whose significance persisted in conventional models—is only an indirect surrogate of hypertrophy, because girth reflects fat, bone, skin and measurement variation as well as muscle cross-sectional area. The larger of the two contributing trials also showed a significant baseline imbalance in exactly this variable, and analysed it with a covariate adjustment that the pooled unadjusted change scores do not reproduce (Section 3.3). The largest estimate is therefore also the least direct and the most vulnerable to residual confounding, which is why it carries the lowest certainty rating. Fourth, publication and small-study bias could not be assessed statistically, because every outcome pooled fewer than ten trials; the extended register search nonetheless identified two completed but unpublished trials on this question, including a 14-week German randomized trial of ashwagandha combined with standardized strength training in strength-trained women and men (DRKS00034276, *n* = 32, completed August 2024), so unpublished evidence bearing directly on the review question is known to exist. Fifth, the subscription databases Embase, Scopus, Web of Science and CINAHL were not searched directly, because institutional subscription access was unavailable to the authors. The revised search instead combined MeSH-augmented PubMed/MEDLINE without date restriction with Europe PMC, OpenAlex, CENTRAL, Semantic Scholar, DHARA, four regional and preprint sources, the ClinicalTrials.gov registry and the WHO ICTRP, together with reference-list, included-study-list and forward-citation screening. CENTRAL, which incorporates trial records supplied from Embase and CINAHL, returned six such records—five Embase-derived and one CINAHL-derived, among them one conference abstract and four reports in Asian and European journals not indexed in MEDLINE—so the content types that a direct Embase or CINAHL search would be expected to add were represented in the screened set. Retrieval bias is thereby reduced, although it cannot be eliminated. Sixth, the review was not prospectively registered in PROSPERO or any other prospective register and no protocol was published in advance (Section 2.1). Seventh, extract type, dose and training prescriptions differed across trials; the lower-body outcome combined leg extension, back squat and leg press, which are direct but not interchangeable measures of lower-body strength; body fat was measured by different methods; and all three trials were manufacturer-funded or manufacturer-supplied. Eighth, a fourth eligible trial could not be entered into the synthesis because its reported strength values were not interpretable on the required scale (Section 3.1 and Section 3.2); that decision rests on data integrity rather than on eligibility, and it means the quantitative synthesis does not exhaust the eligible evidence. Finally, the pooled populations were healthy and predominantly young, so the findings do not extend to older adults or clinical sarcopenia, the settings in which an anti-catabolic adjunct might matter most.

### 4.7. Future Directions

Progress requires adequately powered, independently funded RCTs designed a priori to detect a modest effect (for example, SMD ≈ 0.35 in 1-RM) and pre-registered in a public trial registry, and future syntheses should be registered in PROSPERO and should search the full range of bibliographic databases using both controlled-vocabulary and free-text terms. Such trials should replace girth surrogates with direct measures of hypertrophy—muscle cross-sectional area by magnetic resonance imaging or ultrasound, and lean mass by DEXA—and should standardize and fully disclose the extract, its withanolide content, and the daily dose, alongside a standardized progressive-overload prescription. Because the largest effects were seen in untrained participants, trials in resistance-trained adults and in older adults with or at risk of sarcopenia are the most informative next step, ideally reporting change-score dispersion and individual-level data to permit unbiased pooling. Until such evidence exists, the honest summary is that ashwagandha remains a plausible but unproven adjunct to resistance training.

## 5. Conclusions

Adjunctive ashwagandha combined with structured resistance training was associated with possible benefits in upper- and lower-body maximal strength and in chest girth, but not dependably in body fat, addressing the specific resistance-training question left open by prior performance- and VO_2_max-focused syntheses. These signals nonetheless rest on very few, small, statistically fragile trials, all of which raise at least some concerns on RoB 2; the strength signals are not preserved under small-sample-adjusted inference while the girth estimate could not be meaningfully assessed under that model, and they are of Low certainty for the strength and body-fat outcomes and Very Low certainty for the girth surrogate. They should be read as hypothesis-generating rather than practice-changing. The present evidence therefore does not establish that adjunctive ashwagandha meaningfully enhances resistance-training adaptations. A fourth eligible trial identified during revision could not be pooled because its reported strength values were not interpretable, which further limits what the present evidence can support. Adequately powered, independently funded randomized trials using direct measures of muscle hypertrophy are needed before ashwagandha can be recommended as a resistance-training adjunct. In the meantime, resistance training itself—supported by well-established nutritional co-interventions—remains the evidence-based route to gains in muscle strength and size.

## Figures and Tables

**Table 1:** Characteristics and overall risk of bias of the three randomized controlled trials contributing to the quantitative synthesis.

Study (PMID; Journal)	Design; Country	Participants	Extract; Dose; Duration	Resistance-Training Programme	Outcomes Contributed	RoB 2 Overall
Wankhede 2015 [18] (26609282; *J. Int. Soc. Sports Nutr.*)	Parallel RCT, double-blind; India	57 randomized; 50 analysed (25/25). Healthy men 18–50 y, minimal resistance-training experience	KSM-66 root extract; 600 mg/day (300 mg twice daily); 8 weeks	Supervised progressive resistance training, whole body	Bench 1-RM; leg-extension 1-RM; chest circ.; body fat (BIA)	Some concerns
Ziegenfuss 2018 (STAR) [19] (30463324; *Nutrients*)	Parallel RCT, double-blind; USA	40 randomized; 38 analysed (19/19). Recreationally active men (~26 y)	Sensoril root-and-leaf aqueous extract; 500 mg/day; 12 weeks	Supervised 4-day/week upper/lower split, progressive overload	Bench 1-RM; squat 1-RM; body fat (DEXA)	Some concerns
Verma 2023 [20] (38988644; *F1000Research*)	Parallel RCT, double-blind, multicentre; India	80 randomized; 73 analysed (37/36). Healthy men and women 18–45 y, physically active	KSM-66 root extract; 600 mg/day (300 mg twice daily); 8 weeks	Supervised progressive resistance training, whole body, 3 days/week	Bench 1-RM; leg-press 1-RM; chest circ.; body fat (BIA)	Some concerns *(a)*

*Participant numbers are those analysed for the pooled outcomes.* 1-RM, one-repetition maximum; circ., circumference; BIA, bioelectrical impedance analysis; DEXA, dual-energy X-ray absorptiometry; KSM-66 and Sensoril are standardized commercial extracts; RoB 2, Cochrane Risk of Bias tool, version 2. The fourth eligible trial (Thanawala 2026 [30]) is not listed here because it was reported narratively rather than pooled and was therefore not assigned a RoB 2 rating; its characteristics are given in Section 3.2. *(a) Verma 2023* [20] *was prospectively registered* and reported completely (domain 5 low), but significant baseline imbalance between arms in chest circumference, mid-arm circumference and body-mass index (domain 1) and exclusion of seven randomized participants for poor compliance (domain 3) led to an overall rating of “some concerns”; see Section 3.3.

**Table 2:** Pooled random-effects (REML) change-score standardized mean differences, heterogeneity, and small-sample-adjusted inference by outcome.

Outcome	k	*N*	SMD [95% CI]	*p*	*I*^2^ (%)	95% PI	HKSJ 95% CI	GRADE
1-RM bench press (kg) *(a)*	3	161	0.60 [0.20, 1.01]	0.004	34.3	NE *(c)*	−0.24, 1.45	Low
Lower-body 1-RM (kg) *(a)*	3	161	0.52 [0.19, 0.86]	0.002	6.9	NE *(c)*	−0.20, 1.24	Low
Chest circumference (cm) *(a)*	2	123	0.86 [0.41, 1.31]	<0.001	30.8	NE *(c)*	NE *(d)*	Very Low
Body fat (%) *(b)*	3	161	−0.32 [−0.72, 0.08]	0.120	33.8	NE *(c)*	−1.15, 0.51	Low

SMD, standardized mean difference (Hedges g, change from baseline); k, number of trials; *N*, total participants; CI, confidence interval; PI, prediction interval; NE, not estimated (interval mathematically defined but uninformative at this number of studies; Section 2.7); HKSJ, Hartung–Knapp–Sidik–Jonkman small-sample-adjusted CI. Model: random-effects, restricted maximum likelihood. *(a)* Positive SMD favours ashwagandha. *(b)* Negative SMD favours ashwagandha (greater fat reduction). *(c)* With *k* ≤ 3, τ^2^ is estimated with very low precision and the t multiplier appropriate to a prediction interval (*df* = *k* − 2) is not interpretable; prediction intervals are therefore not reported (Section 2.7). *(d)* At *k* = 2 the HKSJ multiplier is t(0.975, *df* = 1) ≈ 12.71; the interval is mathematically defined but too wide to be informative and is not reported as a meaningful estimate. GRADE certainty was downgraded for risk of bias and imprecision in all outcomes, and additionally for indirectness in chest circumference (Table 3).

**Table 3:** GRADE evidence profile for the effect of adjunctive ashwagandha on resistance-training outcomes.

Outcome (*k*; *N*)	Risk of Bias	Inconsistency	Indirectness	Imprecision	Publ. Bias	Certainty (Interpretation)
1-RM bench press (3; 161)	Serious ⊖	Not serious	Not serious	Serious ⊖	NA †	⊕⊕◯◯ Low—a moderate strength benefit is plausible but fragile.
Lower-body 1-RM (3; 161)	Serious ⊖	Not serious	Not serious ‡	Serious ⊖	NA †	⊕⊕◯◯ Low—benefit plausible; lost on removing Wankhede 2015 [18].
Chest circumference (2; 123)	Serious ⊖	Not serious	Serious ⊖ §	Serious ⊖	NA †	⊕◯◯◯ Very Low—significant in conventional models, but an indirect girth surrogate measured in a trial with baseline imbalance in this variable.
Body fat (3; 161)	Serious ⊖	Not serious	Not serious ¶	Serious ⊖	NA †	⊕⊕◯◯ Low—no significant effect, but unstable; inconclusive.

⊖ indicates a downgrade of one level. Starting certainty is High for randomized trials. In the certainty column, each ⊕ denotes one level of certainty retained and each ◯ one level lost, so that ⊕⊕◯◯ = Low, and ⊕◯◯◯ = Very Low. NA, not assessable. † Publication bias could not be assessed because each outcome pooled fewer than ten trials; funnel-plot and regression-based tests were therefore not performed (Section 2.7). ‡ Lower-body 1-RM combined leg extension, back squat and leg press. All three are direct measures of maximal lower-body strength obtained with standardized 1-RM protocols, so the outcome construct is not indirect; the movement-specific heterogeneity is nevertheless carried to the limitations (Section 4.6). § Chest girth is a surrogate rather than a direct index of muscle cross-sectional area, and baseline chest circumference differed significantly between arms in the larger contributing trial (Section 3.3). ¶ Body fat was measured by DEXA in one trial and by impedance in two; this heterogeneity and the instability in Section 3.6 are discussed but did not further lower an already Low rating.

## Data Availability

All data analysed were extracted from the published included trials and are reported in full in this article and its Supplementary Materials. The complete extraction dataset and the fully scripted analysis pipeline (change-score Hedges g, REML random-effects pooling, leave-one-out, HKSJ, body-fat sensitivity, fixed-effect and figure-generation routines) are provided as Supplementary Materials, so that every estimate reported here can be independently reproduced.

## Supplementary Materials

The following supporting information can be downloaded at: https://www.mdpi.com/article/10.3390/nu18172815/s1, Table S1. Completed PRISMA 2020 checklist. Table S2. Completed PRISMA 2020 for Abstracts checklist. Table S3. Search strategy and record log. (A) Sources searched (final search date 23 August 2026; no date limits applied for any source except the original search); (B) Sources attempted but not accessible, and mitigation; (C) Records supplied to CENTRAL from Embase and CINAHL and screened in this review; (D) Record flow; (E) Reports excluded at full-text assessment (n = 20), with reasons. Table S4. Study-level extraction dataset. (A) Change-from-baseline values as reported in the source publications; (B) Computed effect sizes used in the meta-analysis. Table S5. Sensitivity and robustness analyses. (A) Primary random-effects (REML) estimates and heterogeneity; (B) Leave-one-out analyses; (C) Small-sample-adjusted (HKSJ) and fixed-effect models; (D) Pre-specified body-fat sensitivity analysis; (E) Reporting-bias assessment. File S1. The complete analysis and figure-generation script. The PRISMA 2020 flow diagram is presented as Figure 1 of the main text.
