# Supplementary material for: Adjunctive Ashwagandha (Withania somnifera) Supplementation and Resistance-Training Adaptations in Healthy Adults: A Systematic Review and Random-Effects Meta-Analysis of Muscular Strength, Chest Circumference, and Body Composition
Source: Nutrients. 2026 Aug 27;18(17):2815. doi: 10.3390/nu18172815 (PMC13567585; doi:10.3390/nu18172815)
Supplement: Supplementary file 1 [file nutrients-18-02815-s001.zip › nutrients-4513724-supplementary.pdf]

# Supplementary Materials

**Adjunctive Ashwagandha (*Withania somnifera*) Supplementation and Resistance-Training Adaptations in Healthy Adults: A Systematic Review and Random-Effects Meta-Analysis of Muscular Strength, Muscle Size, and Body Composition**

Jung-Chul Lee and An-Sik Heo — Department of Sports Medicine, Dongshin University, Naju 58245, Republic of Korea

**Contents.** Table S1, completed PRISMA 2020 checklist; Table S2, completed PRISMA 2020 for Abstracts checklist; Table S3, search strategy and record log; Table S4, study-level extraction dataset; Table S5, sensitivity and robustness analyses; File S1, analysis script (provided as a separate file). The PRISMA 2020 flow diagram is presented as Figure 1 of the main manuscript.

## Table S1. PRISMA 2020 Checklist

Adapted from Page MJ, McKenzie JE, Bossuyt PM, et al. The PRISMA 2020 statement: an updated guideline for reporting systematic reviews. *BMJ* 2021;372:n71. "Location where item is reported" gives the numbered section of the manuscript; section numbers are used in preference to page numbers because pagination changes at typesetting.

| Section and Topic | Item # | Checklist item | Location where item is reported |
|---|---|---|---|
| **TITLE** | | | |
| **Title** | 1 | Identify the report as a systematic review. | Title page — the title states "A Systematic Review and Random-Effects Meta-Analysis" |
| **ABSTRACT** | | | |
| **Abstract** | 2 | See the PRISMA 2020 for Abstracts checklist. | Abstract; see Table S2 |
| **INTRODUCTION** | | | |
| **Rationale** | 3 | Describe the rationale for the review in the context of existing knowledge. | Section 1, paragraphs 1–3 |
| **Objectives** | 4 | Provide an explicit statement of the objective(s) or question(s) the review addresses. | Section 1, final paragraph; Section 2.1 (PICO) |
| **METHODS** | | | |
| **Eligibility criteria** | 5 | Specify the inclusion and exclusion criteria for the review and how studies were grouped for the syntheses. | Sections 2.2 and 2.3 |
| **Information sources** | 6 | Specify all databases, registers, websites, organisations, reference lists and other sources searched or consulted, and the date when each source was last searched or consulted. | Section 2.4; Table S3 |
| **Search strategy** | 7 | Present the full search strategies for all databases, registers and websites, including any filters and limits used. | Section 2.4 (full Boolean strings for all twelve databases and two registers, dates, limits and fields); Table S3, Panels A and B |
| **Selection process** | 8 | Specify the methods used to decide whether a study met the inclusion criteria, including how many reviewers screened each record and each report retrieved, whether they worked independently, and if applicable, details of automation tools used. | Section 2.5 (two reviewers, independently and in duplicate; disagreements resolved by consensus; no automation tools used) |
| **Data collection process** | 9 | Specify the methods used to collect data from reports, including how many reviewers collected data from each report, whether they worked independently, any processes for obtaining or confirming data from study investigators, and if applicable, details of automation tools used. | Section 2.5 (two reviewers, independently and in duplicate; values read verbatim from published tables; study investigators were not contacted; no automation tools used) |
| **Data items** | 10a | List and define all outcomes for which data were sought. Specify whether all results that were compatible with each outcome domain in each study were sought, and if not, the methods used to decide which results to collect. | Section 2.3 |
| | 10b | List and define all other variables for which data were sought. Describe any assumptions made about any missing or unclear information. | Section 2.5; Section 2.6; Table 1; Table S4 |
| **Study risk of bias assessment** | 11 | Specify the methods used to assess risk of bias in the included studies, including details of the tool(s) used, how many reviewers assessed each study and whether they worked independently, and if applicable, details of automation tools used. | Section 2.8 (Cochrane RoB 2; both authors independently; consensus; no automation tools used) |
| **Effect measures** | 12 | Specify for each outcome the effect measure(s) used in the synthesis or presentation of results. | Section 2.6 (change-from-baseline standardised mean difference, Hedges g) |

| Section and Topic | Item # | Checklist item | Location where item is reported |
|---|---|---|---|
| **Synthesis methods** | 13a | Describe the processes used to decide which studies were eligible for each synthesis. | Sections 2.3 and 3.1 |
| | 13b | Describe any methods required to prepare the data for presentation or synthesis, such as handling of missing summary statistics, or data conversions. | Section 2.5 (algebraic recovery of change-score SD from a reported standard error); Section 2.6 |
| | 13c | Describe any methods used to tabulate or visually display results of individual studies and syntheses. | Section 2.7; Figures 1–3; Tables 1–3; Tables S4 and S5 |
| | 13d | Describe any methods used to synthesise results and provide a rationale for the choice(s). If meta-analysis was performed, describe the model(s), method(s) to identify the presence and extent of statistical heterogeneity, and software package(s) used. | Section 2.7 (random-effects model, REML estimator of tau-squared; Cochran Q and I-squared; scripted pipeline released as File S1) |
| | 13e | Describe any methods used to explore possible causes of heterogeneity among study results. | Sections 2.7 and 3.5 |
| | 13f | Describe any sensitivity analyses conducted to assess robustness of the synthesised results. | Section 2.7 (leave-one-out; Hartung–Knapp–Sidik–Jonkman; fixed-effect refit; pre-specified body-fat exclusion); Sections 3.5 and 3.6; Table S5 |
| **Reporting bias assessment** | 14 | Describe any methods used to assess risk of bias due to missing results in a synthesis (arising from reporting biases). | Section 2.7 — funnel-plot asymmetry and regression-based tests were not performed because every synthesis pooled fewer than ten studies; the justification and supporting references are given |
| **Certainty assessment** | 15 | Describe any methods used to assess certainty (or confidence) in the body of evidence for an outcome. | Section 2.8 (GRADE) |
| RESULTS | | | |
| **Study selection** | 16a | Describe the results of the search and selection process, from the number of records identified in the search to the number of studies included in the review, ideally using a flow diagram. | Section 3.1; **Figure 1 (PRISMA 2020 flow diagram)** |
| | 16b | Cite studies that might appear to meet the inclusion criteria, but which were excluded, and explain why they were excluded. | Section 3.1 and Table S3, Panel E (twenty full-text reports excluded, each with its reason) |
| **Study characteristics** | 17 | Cite each included study and present its characteristics. | Section 3.2; Table 1 |
| **Risk of bias in studies** | 18 | Present assessments of risk of bias for each included study. | Section 3.3; Figure 3; Table 1 |
| **Results of individual studies** | 19 | For all outcomes, present, for each study: (a) summary statistics for each group (where appropriate) and (b) an effect estimate and its precision, ideally using structured tables or plots. | Figure 2 (forest plots); Table S4 (Panels A and B) |
| **Results of syntheses** | 20a | For each synthesis, briefly summarise the characteristics and risk of bias among contributing studies. | Sections 3.2 and 3.3 |
| | 20b | Present results of all statistical syntheses conducted. If meta-analysis was done, present for each the summary estimate and its precision and measures of statistical heterogeneity. If comparing groups, describe the direction of the effect. | Section 3.4; Table 2; Figure 2 |
| | 20c | Present results of all investigations of possible causes of heterogeneity among study results. | Section 3.5; Table S5, Panel A |
| | 20d | Present results of all sensitivity analyses conducted to assess the robustness of the synthesised results. | Sections 3.5 and 3.6; Table S5, Panels B–D |
| **Reporting biases** | 21 | Present assessments of risk of bias due to missing results (arising from reporting biases) for each synthesis assessed. | Section 3.6 — not assessable; every synthesis pooled fewer than ten studies. Reflected in Table 3 (footnote †) and Table S5, Panel E |
| **Certainty of evidence** | 22 | Present assessments of certainty (or confidence) in the body of evidence for each outcome assessed. | Section 3.7; Table 3 (GRADE evidence profile) |
| DISCUSSION | | | |
| **Discussion** | 23a | Provide a general interpretation of the results in the context of other evidence. | Sections 4.1, 4.2 and 4.3 |
| | 23b | Discuss any limitations of the evidence included in the review. | Section 4.6 |
| | 23c | Discuss any limitations of the review processes used. | Section 4.6 (single primary database; free-text search without controlled vocabulary; no prospective registration) |
| | 23d | Discuss implications of the results for practice, policy, and future research. | Sections 4.4 and 4.7 |

| Section and Topic | Item # | Checklist item | Location where item is reported |
|---|---|---|---|
| **OTHER INFORMATION** | | | |
| **Registration and protocol** | 24a | Provide registration information for the review, including register name and registration number, or state that the review was not registered. | **Section 2.1 — the review was not registered in PROSPERO or in any other prospective register**; also stated in Section 4.6 |
| | 24b | Indicate where the review protocol can be accessed, or state that a protocol was not prepared. | **Section 2.1 — no protocol was published or made publicly available**; an internal, version-controlled analysis plan is released with File S1 |
| | 24c | Describe and explain any amendments to information provided at registration or in the protocol. | Section 2.1 — no amendments were made to the pre-specified analysis plan. One addition was made to the eligibility framework at the second revision: a data-integrity condition under which an eligible trial whose reported outcome values are incompatible with the stated measurement scale is reported narratively rather than pooled. This condition is identified as post hoc in Section 2.2 and was applied to one trial (Section 3.2) |
| **Support** | 25 | Describe sources of financial or non-financial support for the review, and the role of the funders or sponsors in the review. | Funding statement — no external funding was received; also stated in the Abstract |
| **Competing interests** | 26 | Declare any competing interests of review authors. | Conflicts of Interest statement |
| **Availability of data, code and other materials** | 27 | Report which of the following are publicly available and where they can be found: template data collection forms; data extracted from included studies; data used for all analyses; analytic code; any other materials used in the review. | Data Availability Statement; Table S3 (search log); Table S4 (extracted data and data used in all analyses); Table S5 (analysis output); File S1 (analytic code) |

## Table S2. PRISMA 2020 for Abstracts Checklist

| Section and Topic | Item # | Checklist item | Reported |
|---|---|---|---|
| **TITLE** | | | |
| **Title** | 1 | Identify the report as a systematic review. | Yes — the title states "A Systematic Review and Random-Effects Meta-Analysis" |
| **BACKGROUND** | | | |
| **Objectives** | 2 | Provide an explicit statement of the main objective(s) or question(s) the review addresses. | Yes — Abstract, Background/Objectives (final sentence) |
| **METHODS** | | | |
| **Eligibility criteria** | 3 | Specify the inclusion and exclusion criteria for the review. | Yes — Abstract, Methods (randomised controlled trials combining a standardised ashwagandha root extract with structured resistance training in healthy adults) |
| **Information sources** | 4 | Specify the information sources and the date when each was last searched. | Yes — Abstract, Methods (PubMed/MEDLINE, 1 January 2010 to 8 July 2026; reference-list and citation screening) |
| **Risk of bias** | 5 | Specify the methods used to assess risk of bias in the included studies. | Yes — Abstract, Methods (Cochrane RoB 2) |
| **Synthesis of results** | 6 | Specify the methods used to present and synthesise results. | Yes — Abstract, Methods (change-from-baseline Hedges g pooled with restricted maximum-likelihood random-effects models; leave-one-out and HKSJ sensitivity analyses; GRADE) |
| **RESULTS** | | | |
| **Included studies** | 7 | Give the total number of included studies and participants and summarise relevant characteristics. | Yes — Abstract, Results (four eligible RCTs; three pooled, n = 161; one reported narratively) |
| **Synthesis of results** | 8 | Present results for main outcomes, preferably indicating the number of included studies and participants for each. If meta-analysis was done, report the summary estimate and confidence interval. If comparing groups, indicate the direction of the effect. | Yes — Abstract, Results (summary SMD with 95% CI for all four outcomes; direction of effect stated; heterogeneity reported) |
| **DISCUSSION** | | | |
| **Limitations of evidence** | 9 | Provide a brief summary of the limitations of the evidence included in the review. | Yes — Abstract, Results and Conclusions (risk of bias in all three trials; loss of significance under HKSJ; dependence on single influential trials; GRADE Low to Very Low) |

| Section and Topic | Item # | Checklist item | Reported |
|---|---|---|---|
| **Interpretation** | 10 | Provide a general interpretation of the results and important implications. | Yes — Abstract, Conclusions |
| **OTHER** | | | |
| **Funding** | 11 | Specify the primary source of funding for the review. | Yes — Abstract, Conclusions (final sentence: no external funding) |
| **Registration** | 12 | Provide the register name and registration number. | Yes — Abstract, Conclusions (final sentence: the review was not registered) |

# Table S3. Search Strategy and Record Log

### Panel A. Sources searched (final search date 23 August 2026; no date limits applied at any source except the original search)

| Source | Interface / platform | Date last searched | Date limits | Controlled vocabulary / fields | Records |
|---|---|---|---|---|---|
| PubMed / MEDLINE (original search) | pubmed.ncbi.nlm.nih.gov | 8 Jul 2026 | 1 Jan 2010 – 8 Jul 2026 | Title/abstract free text only | 29 |
| PubMed / MEDLINE (re-run, MeSH-augmented) | NCBI E-utilities | 22 Aug 2026 | None | "Withania"[Mesh]; "Resistance Training"[Mesh]; "Muscle Strength"[Mesh] + ti/ab | 31 |
| Europe PMC (including preprints) | EBI Europe PMC REST | 22 Aug 2026 | None | TITLE_ABS | 27 |
| OpenAlex | api.openalex.org | 22 Aug 2026 | None | title_and_abstract.search | 89 |
| Cochrane CENTRAL (Trials) | cochranelibrary.com | 23 Aug 2026 | None | ti,ab,kw + MeSH descriptors | 51 |
| Semantic Scholar (bulk search) | api.semanticscholar.org/graph/v1 | 23 Aug 2026 | None | Title/abstract | 748 |
| Semantic Scholar (forward citations of the three pooled trials) | api.semanticscholar.org/graph/v1 | 23 Aug 2026 | None | Citation graph | 263 |
| DHARA (Digital Helpline for Ayurveda Research Articles) | dharaonline.org | 23 Aug 2026 | None | Keyword | 266 |
| J-STAGE | jstage.jst.go.jp | 23 Aug 2026 | None | Keyword | 203 |
| KoreaMed | koreamed.org | 23 Aug 2026 | None | Keyword | 12 |
| medRxiv | api.biorxiv.org | 23 Aug 2026 | None | Full record scan | 16 |
| bioRxiv | api.biorxiv.org | 23 Aug 2026 | None | Full record scan | 49 |
| CORE | api.core.ac.uk (v3) | 23 Aug 2026 | None | Keyword | 6 |
| J Int Soc Sports Nutr conference abstracts | PubMed, journal-limited | 23 Aug 2026 | None | Journal + keyword | 1 |
| **Subtotal, databases** | | | | | **1,762** |
| ClinicalTrials.gov (register) | clinicaltrials.gov API v2 | 22 Aug 2026 | None | Condition / intervention | 81 |
| WHO ICTRP (register) | trialsearch.who.int | 23 Aug 2026 | None | Four queries merged; includes 411 records from the Clinical Trials Registry–India | 498 |
| **Subtotal, registers** | | | | | **579** |
| **Total records identified** | | | | | **2,341** |

Concept A (substance): ashwagandha OR "Withania somnifera" OR withanolide* OR "Indian ginseng" OR ashvagandha OR asvagandha OR "winter cherry" OR KSM-66 OR Sensoril OR Shoden. Concept B (exercise / outcome): "resistance training" OR "strength training" OR "resistance exercise" OR "weight training" OR "weight lifting" OR "muscle strength" OR hypertrophy OR "body composition" OR "1-RM" OR "one repetition maximum" OR "muscle size" OR "lean body mass" OR "fat free mass" OR "muscle mass" OR "physical training" OR exercise. Final query = Concept A AND Concept B, adapted to the field syntax of each interface. No language, publication-type or study-design filter was applied at the search stage; design and population eligibility were applied at screening.

### Panel B. Sources attempted but not accessible, and mitigation

| Source | Reason not searched | Mitigation |
|---|---|---|
| Embase (Elsevier) | Institutional subscription required; the search interface returned a login-only application shell | Embase-derived trial records were retrieved indirectly through CENTRAL (five records; Panel C) |
| CINAHL (EBSCOhost) | Institutional authentication required; the authentication | CINAHL-derived trial record retrieved |

| | | |
|---|---|---|
| | chain terminated at the identity-provider callback | indirectly through CENTRAL (one record; Panel C) |
| Scopus (Elsevier) | Institutional subscription required | Coverage partly substituted by OpenAlex, Semantic Scholar and CORE |
| Web of Science (Clarivate) | Institutional subscription required | Coverage partly substituted by OpenAlex, Semantic Scholar and CORE |
| Clinical Trials Registry–India (direct interface) | Advanced-search form requires CAPTCHA completion | 411 CTRI records retrieved through the WHO ICTRP |
| AYUSH Research Portal | Server not reachable during the search window | DHARA searched instead (266 records) |
| Lens.org; Dimensions.ai | Account or browser session required for retrieval | Coverage overlaps OpenAlex and Semantic Scholar |
| BASE; SciELO | Automated retrieval blocked by the provider | Coverage partly substituted by CORE and OpenAlex |
| Shodhganga; NDLTD; DART-Europe | Service unavailable or discontinued | No substitute; recorded as a limitation (Section 4.6) |
| Google Scholar | Automated retrieval not attempted, as it is contrary to the terms of service | Search URLs recorded; results not machine-harvested |

**Panel C. Records supplied to CENTRAL from Embase and CINAHL and screened in this review**

| **Record (journal, year)** | **Source database** |
|---|---|
| Efficacy of ayurvedic herb Withania somnifera in management of muscle sarcopenia — Annals of Neurology, 2018 (conference abstract) | Embase |
| Efficacy of ashwagandha in improving cardiorespiratory endurance (VO2 max test) in healthy subjects — International Journal of Research in Pharmaceutical Sciences, 2021 | Embase |
| Effect of selected yoga techniques along with ashwagandha and vacha in depression — Asian Journal of Pharmaceutical and Clinical Research, 2021 | Embase |
| Effects of eight-week supplementation of ashwagandha on cardiorespiratory endurance in elite Indian cyclists — Journal of Ayurveda and Integrative Medicine, 2012 | Embase |
| Ashwagandha root extract stabilises physiological stress responses in male and female academy athletes during pre-season training — International Journal of Sport Nutrition and Exercise Metabolism, 2026 | Embase |
| The role of Withania somnifera supplementation in modulating physical and physiological performance responses to regular football training — Fizjoterapia Polska, 2024 | CINAHL |

These six records comprise one conference abstract and five reports in Asian and European journals not indexed in MEDLINE — the content types that a direct Embase or CINAHL search would be expected to contribute. All six were screened and none met the eligibility criteria.

**Panel D. Record flow**

| **Stage** | **n** |
|---|---|
| Records identified from databases | 1,762 |
| Records identified from registers | 579 |
| Duplicate records removed before screening | 426 |
| Records removed by automation tools | 0 (no automation tools were used) |
| Records screened | 1,915 |
| Records excluded at title/abstract — database records | 1,362 |
| Records excluded at title/abstract — register entries with no eligible published report | 529 |
| Reports sought for retrieval | 24 |
| Reports not retrieved | 0 |
| Reports assessed for eligibility in full text | 24 |
| Reports excluded at full text | 20 (Panel E) |
| Studies included in the systematic review | 4 |
| Studies included in the quantitative synthesis | 3 — Wankhede 2015 [18]; Ziegenfuss (STAR) 2018 [19]; Verma 2023 [20] |
| Studies included in the review but reported narratively, not pooled | 1 — Thanawala 2026 [70] (see Section 3.2) |
| Additional eligible records from reference lists, prior-review included-study lists and forward citations | 0 |

Cross-checking covered the included-study lists of the four prior syntheses [21–24] and of one further 2026 meta-analysis identified during this search. Eight trials appearing in those lists had not previously been assessed by us; all eight proved ineligible. The register search additionally identified two completed but unpublished trials relevant to this question, including DRKS00034276 (Germany; ashwagandha combined with standardized strength training in strength-trained women and men; n = 32; completed August 2024), which is discussed in Section 4.6.

**Panel E. Reports excluded at full-text assessment (n = 20), with reasons**

| # | Report | Reason for exclusion | Criterion |
|---|---|---|---|
| 1 | High-intensity interval training trial [49] | No structured resistance-training co-intervention | Intervention |
| 2 | Team-sport pre-season stress trial [50] | No structured resistance-training co-intervention; competitive athletes | Intervention |
| 3 | Enriched-milk sleep trial [51] | No resistance-training co-intervention; combination product | Intervention |
| 4 | Acute-recovery trial (University of Saskatchewan Undergraduate Research Journal, 2025) | Acute post-exercise recovery; no training programme delivered | Intervention |
| 5 | Conference report of self-directed training (2020) | Training not prescribed or supervised by the investigators | Intervention |
| 6 | Effects of ashwagandha supplementation on physical performance (2019) | Supplementation without a prescribed training programme | Intervention |
| 7 | Efficacy and safety of low-dose ashwagandha on exercise endurance (2026) | Supplementation without a prescribed training programme | Intervention |
| 8 | Efficacy of ashwagandha in improving cardiorespiratory endurance, VO2 max test (Int J Res Pharm Sci, 2021) | Supplementation without a prescribed training programme | Intervention |
| 9 | Female athletes, perception of recovery and muscle strength [54] | Competitive athletes; non-standardized training; non-poolable strength measure | Population |
| 10 | Wrestlers, body composition and blood health (Pol J Sport Tourism, 2023) | Competitive athletes | Population |
| 11 | Handball players, eight-day supplementation (Nutr Health, 2026) | Competitive athletes | Population |
| 12 | National-team wrestlers, special endurance (2025) | Competitive athletes | Population |
| 13 | Elite Indian cyclists, anaerobic performance (Medicina Sportiva, 2012) | Competitive athletes (state-level medallists); no resistance-training co-intervention; withanolide content not stated | Population |
| 14 | Cycle-ergometer performance trial [53] | Performance testing rather than a training programme; no poolable outcome | Outcome |
| 15 | Serum testosterone and muscle recovery in strength training (Int J Med Pharm Res, 2023) | Companion report; hormonal and inflammatory outcomes only | Outcome |
| 16 | Raut, A.A.; Rege, N.N.; Tadvi, F.M.; Solanki, P.V.; Kene, K.R.; Shirolkar, S.G.; Pandey, S.N.; Vaidya, R.A. Exploratory study to evaluate tolerability, safety, and activity of Ashwagandha (Withania somnifera) in healthy volunteers. J. Ayurveda Integr. Med. 2012, 3, 111–114 | No strength or body-composition outcome on a poolable scale | Outcome |
| 17 | Crossover trial in ageing overweight males [52] | Crossover design; no exercise co-intervention | Design |
| 18 | Acute dose-response report (2026) | Crossover design | Design |
| 19 | Pastina, J.T.; Stewart, L.K. Nootropic supplements and exercise: Patterns and perceptions of cognitive enhancer use in physically active individuals. J. Diet. Suppl. 2026, 23, 23–41 | Not a randomized controlled trial | Design |
| 20 | Root-powder supplementation and core muscle strength (2014) | Non-standardized preparation; withanolide content not stated | Intervention |

Criterion codes refer to the eligibility criteria in Section 2.2 of the manuscript. Criterion codes for entries 16 and 19 refer to the outcome and design criteria respectively; both were excluded during the original screening round.

The corresponding PRISMA 2020 flow diagram, showing these numbers at each stage, is presented as Figure 1 of the main manuscript. The original PubMed/MEDLINE string reproduces the 29 retrieved records exactly when run with the stated date limits, and returns the identical set when re-run with no date limit, confirming that the 2010 restriction excluded no record (Section 2.2).

## Table S4. Study-Level Extraction Dataset

### Panel A. Change-from-baseline values as reported in the source publications

| Outcome | Study | Arm | n | Mean change | SD of change | Source |
|---|---|---|---|---|---|---|
| 1-RM bench press (kg) | Wankhede 2015 | Ashwagandha | 25 | 46.05 | 23.00 | Table 2 |
| | | Placebo | 25 | 26.42 | 16.72 | Table 2 |
| | Ziegenfuss 2018 | Ashwagandha | 19 | 12.8 | 8.20 | Table 3 |

| Outcome | Study | Arm | n | Mean change | SD of change | Source |
|---|---|---|---|---|---|---|
| | | Placebo | 19 | 8.0 | 6.00 | Table 3 |
| | Verma 2023 | Ashwagandha | 37 | 11.0 | 7.30 | Table 3 |
| | | Placebo | 36 | 8.9 | 6.30 | Table 3 |
| Lower-body 1-RM (kg) | Wankhede 2015 (leg extension) | Ashwagandha | 25 | 14.50 | 9.04 | Table 2 |
| | | Placebo | 25 | 9.77 | 6.27 | Table 2 |
| | Ziegenfuss 2018 (back squat) | Ashwagandha | 19 | 19.1 | 13.00 | Table 3 |
| | | Placebo | 19 | 10.0 | 6.20 | Table 3 |
| | Verma 2023 (leg press) | Ashwagandha | 37 | 7.0 | 9.03 | Table 3 |
| | | Placebo | 36 | 4.5 | 8.20 | Table 3 |
| Chest circumference (cm) | Wankhede 2015 | Ashwagandha | 25 | 3.37 | 1.89 | Table 3 |
| | | Placebo | 25 | 1.43 | 1.45 | Table 3 |
| | Verma 2023 | Ashwagandha | 37 | 3.6 | 1.90 | Table 3 |
| | | Placebo | 36 | 2.5 | 1.30 | Table 3 |
| Body fat (%) | Wankhede 2015 | Ashwagandha | 25 | −3.47 | 3.58 | Table 4 |
| | | Placebo | 25 | −1.52 | 2.58 | Table 4 |
| | Ziegenfuss 2018 | Between-group difference | 19 / 19 | −0.80 | SE 0.57 → SD 1.76 ‡ | Table 4 |
| | Verma 2023 | Ashwagandha | 37 | −1.7 | 0.30 | Table 3 |
| | | Placebo | 36 | −1.7 | 0.30 | Table 3 |

All values are the change-from-baseline means and standard deviations printed in the source tables, with the single exception marked ‡ below; no value was imputed from a figure, median or range, and no value was estimated from a study without the required statistic. ‡ For the STAR trial the body-fat change-score SD was recovered algebraically from the reported standard error of the between-group difference using $SD = SE_{diff} \times \sqrt{n/2}$, as described in Section 2.5 of the manuscript. Body-fat change scores are shown as reductions (negative values) so that the direction of effect is consistent across outcomes.

### Panel B. Computed effect sizes used in the meta-analysis

| Outcome | Study | Hedges *g* | 95% CI | Variance | Random-effects weight (%) |
|---|---|---|---|---|---|
| 1-RM bench press | Wankhede 2015 | 0.96 | 0.38 to 1.55 | 0.0893 | 31.3 |
| | Ziegenfuss 2018 | 0.65 | 0.00 to 1.31 | 0.1109 | 27.1 |
| | Verma 2023 | 0.30 | −0.16 to 0.77 | 0.0554 | 41.6 |
| Lower-body 1-RM | Wankhede 2015 | 0.60 | 0.03 to 1.17 | 0.0836 | 31.4 |
| | Ziegenfuss 2018 | 0.87 | 0.21 to 1.54 | 0.1153 | 23.4 |
| | Verma 2023 | 0.29 | −0.17 to 0.75 | 0.0554 | 45.2 |
| Chest circumference | Wankhede 2015 | 1.13 | 0.54 to 1.73 | 0.0929 | 42.0 |
| | Verma 2023 | 0.67 | 0.20 to 1.14 | 0.0579 | 58.0 |
| Body fat | Wankhede 2015 | −0.62 | −1.18 to −0.05 | 0.0838 | 31.9 |
| | Ziegenfuss 2018 | −0.45 | −1.09 to 0.20 | 0.1079 | 26.9 |
| | Verma 2023 | 0.00 | −0.46 to 0.46 | 0.0548 | 41.1 |

Hedges *g* is the between-group difference in mean change divided by the pooled change-score SD, with the small-sample correction $J = 1 - 3/(4\cdot df - 1)$. Random-effects weights are $1/(v_i + \tau^2)$ normalised to 100%, using the REML estimate of $\tau^2$ for each outcome. Every value in this panel, and every pooled estimate in Table S5, is reproduced exactly by File S1 from the Panel A inputs alone.

## Table S5. Sensitivity and Robustness Analyses

### Panel A. Primary random-effects (REML) estimates and heterogeneity

| Outcome | k | N | SMD [95% CI] | p | $I^2$ (%) | $\tau^2$ | Q (p) |
|---|---|---|---|---|---|---|---|
| 1-RM bench press | 3 | 161 | 0.60 [0.20, 1.01] | 0.004 | 34.3 | 0.048 | 3.05 (0.22) |
| Lower-body 1-RM | 3 | 161 | 0.52 [0.19, 0.86] | 0.002 | 6.9 | 0.009 | 2.15 (0.34) |
| Chest circumference | 2 | 123 | 0.86 [0.41, 1.31] | < 0.001 | 30.8 | 0.034 | 1.45 (0.23) |

| Outcome | k | N | SMD [95% CI] | p | I² (%) | τ² | Q (p) |
|---|---|---|---|---|---|---|---|
| Body fat | 3 | 161 | −0.32 [−0.72, 0.08] | 0.120 | 33.8 | 0.046 | 3.02 (0.22) |

## Panel B. Leave-one-out analyses

| Outcome | Study omitted | Pooled SMD [95% CI] | Remains significant? |
|---|---|---|---|
| 1-RM bench press | Wankhede 2015 | 0.42 [0.04, 0.80] | Yes |
| | Ziegenfuss 2018 | 0.61 [−0.03, 1.25] | No |
| | Verma 2023 | 0.82 [0.39, 1.26] | Yes |
| Lower-body 1-RM | Wankhede 2015 | 0.53 [−0.04, 1.10] | No |
| | Ziegenfuss 2018 | 0.41 [0.05, 0.77] | Yes |
| | Verma 2023 | 0.71 [0.28, 1.15] | Yes |
| Chest circumference | Wankhede 2015 | 0.67 [0.20, 1.14] | Yes |
| | Verma 2023 | 1.13 [0.54, 1.73] | Yes |
| Body fat | Wankhede 2015 | −0.16 [−0.58, 0.26] | No |
| | Ziegenfuss 2018 | −0.28 [−0.89, 0.32] | No |
| | Verma 2023 | −0.54 [−0.97, −0.12] | Yes |

Omissions not quoted in the main text are reported here for completeness. All values are reproduced by File S1.

## Panel C. Small-sample-adjusted (HKSJ) and fixed-effect models

| Outcome | Random-effects (REML) | HKSJ 95% CI | Significant under HKSJ? | Fixed-effect SMD |
|---|---|---|---|---|
| 1-RM bench press | 0.60 [0.20, 1.01] | −0.24 to 1.45 | No | 0.58 |
| Lower-body 1-RM | 0.52 [0.19, 0.86] | −0.20 to 1.24 | No | 0.51 |
| Chest circumference | 0.86 [0.41, 1.31] | Not reported (k = 2; interval uninformative) | Not assessable | 0.85 |
| Body fat | −0.32 [−0.72, 0.08] | −1.15 to 0.51 | No | −0.29 |

At $k = 2$ the HKSJ multiplier is the 97.5th percentile of the t-distribution on one degree of freedom ($\approx 12.71$), which renders the interval uninterpretable; it is therefore reported as not estimable. Prediction intervals were not estimable for any outcome, because with $k \leq 3$ the t multiplier appropriate to a prediction interval has $df = k - 2 = 1$ (Section 2.7).

## Panel D. Pre-specified body-fat sensitivity analysis

| Analysis | k | SMD [95% CI] | p | I² (%) |
|---|---|---|---|---|
| All trials | 3 | −0.32 [−0.72, 0.08] | 0.120 | 33.8 |
| Excluding Verma 2023 (reported change-score SD of 0.3% judged implausibly precise) | 2 | −0.54 [−0.97, −0.12] | 0.013 | 0.0 |

## Panel E. Reporting-bias assessment

Funnel-plot asymmetry and regression-based tests for small-study effects, including the Egger test, were not performed for any outcome, because each synthesis pooled fewer than ten trials — a number at which such tests are uninformative. Publication bias could therefore not be assessed. This is reflected in the GRADE evidence profile (Table 3, footnote †), in the Results (Section 3.6) and in the limitations (Section 4.6), and corresponds to PRISMA 2020 items 14 and 21.
